# Phosphorylation at the stigmatic gate: how FERONIA regulates actin organization to control pollen hydration

**DOI:** 10.1093/plphys/kiag137

**Published:** 2026-03-30

**Authors:** Jessy Silva

**Affiliations:** Assistant Features Editor, Plant Physiology, American Society of Plant Biologists; LAQV/REQUIMTE, Department of Biology, Faculty of Sciences, University of Porto, Porto, Portugal

In flowering plants, sexual reproduction begins with pollination, when pollen is transferred from the anther to the stigma. Pollen grains are released in a dehydrated state and must absorb water from the stigmatic papilla cells to reactivate metabolism, germinate, and form a pollen tube that transports sperm cells through the pistil to the ovule, where double fertilization gives rise to a new seed (Chapman and Goring 2010; Dresselhaus and Franklin-Tong 2013; He et al. 2025). This initial pollen hydration step is tightly regulated by stigmatic cells through not-fully understood mechanisms referred to as the stigmatic gate, which determines whether pollen is allowed to hydrate, germinate, and fertilize.

In nature, stigmas are exposed to pollen from multiple sources, including the same plant, other individuals of the same species, and closely related species (Huang et al. 2023). Many flowering plants have evolved self-incompatibility systems to prevent self-fertilization, promote outcrossing, and enhance genetic diversity. In these species, stigmatic cells recognize and reject pollen from the same plant (self-pollen) while allowing pollen from other plants to hydrate. As a result, self-pollen is incompatible and rejected, whereas non-self pollen is compatible and accepted (Iwano et al. 2007; Chapman and Goring 2010).

Members of the Brassicaceae family, including the model plant *Arabidopsis thaliana*, have a dry stigma lacking surface secretions. Dry stigmas are highly selective, accepting and hydrating compatible pollen while rejecting incompatible pollen, a pollen-stigma recognition process via complex peptide-receptor signaling (Chapman and Goring 2010; He et al. 2025). Several regulatory molecules have been identified that are involved in the recognition of compatible pollen. In Arabidopsis stigmatic cells, the receptor-like kinase FERONIA (FER) acts as a gatekeeper of the stigmatic gate controlling pollen hydration. Prior work established that FER perceives RAPID ALKALINIZATION FACTOR (RALF) peptides, such as RALF33, secreted by the stigma, which function as negative regulators to maintain the stigmatic gate closed and restrict pollen hydration (Fig. 1a). Upon pollination, POLLEN COAT PROTEIN B-class (PCP-B) peptides from the pollen wall compete with RALFs to bind FER, relieving this restriction, opening the sigmatic gate, and allowing compatible pollen to hydrate (Fig. 1a) (Liu et al. 2021).

**Figure 1:**
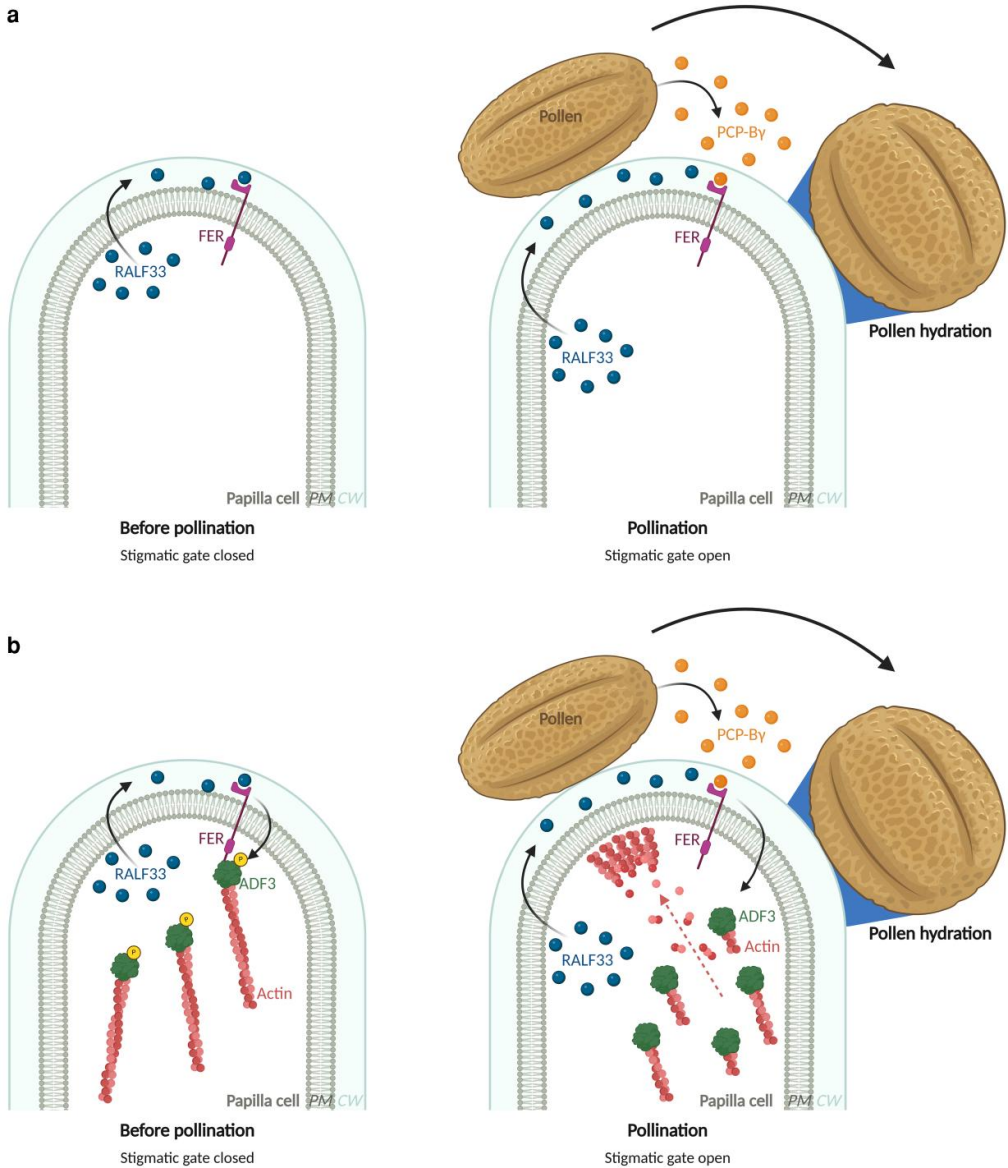
Stigmatic gate model controlling pollen hydration. **a)** Model prior to this study. Before pollination, FERONIA (FER) senses RALF33 peptides from papilla cells, which act as negative regulators to restrict pollen hydration and maintain the stigmatic gate closed. Upon pollination, PCP-Bγ peptides secreted by pollen compete with RALF33 for FER binding, opening the stigmatic gate and facilitating pollen hydration. **b)** Updated model incorporating new findings. Before pollination, stigma-derived RALF33 binds FER, promoting phosphorylation of ADF3 and inhibiting ADF3's actin-depolymerizing activity, maintaining a stabilized actin network and keeping the stigmatic gate closed. Upon pollination, pollen-derived PCP-Bγ peptides compete with RALF33 for FER binding. Binding of PCP-Bγ to FER antagonizes RALF33–FER signaling, activates ADF3's actin-depolymerizing activity, promotes actin accumulation at the pollen contact site, and opens the stigmatic gate for pollen hydration. Created in https://BioRender.com.

Actin cytoskeleton plays a central role in pollen-stigma recognition. Compatible pollination induces actin reorganization and polymerization and accumulation at the pollen contact site in papilla cells, whereas incompatible pollination triggers actin depolymerization (Iwano et al. 2007; Rozier et al. 2020). Actin dynamics are regulated by actin-depolymerizing factors (ADFs), which can be inactivated by kinase-mediated phosphorylation to modulate filament organization (Lu et al. 2020; Wang et al. 2023).

As yet, the connection between the FERONIA receptor, actin reorganization, and pollen hydration remains unclear. In this issue of *Plant Physiology*, He et al. (2026) used live-imaging to demonstrate that pollen hydration depends on actin remodeling in Arabidopsis stigmatic cells, with actin accumulating at the pollen contact site. Inhibition of actin polymerization with Latrunculin B blocked this accumulation and slowed pollen hydration. Furthermore, analysis of *fer* mutants revealed earlier actin accumulation at the pollen contact site, suggesting that FER controls actin remodeling timing.

Through a protein-protein interaction analysis, He et al. found that FER physically interacts with actin-depolymerizing factor ADF3, which is expressed in the stigma. In *adf3* mutants, actin failed to accumulate at the pollen contact site, slowing pollen hydration rate, a defect rescued by reintroducing *ADF3*. The double mutants *fer adf3* and *ralf33 adf3* showed slower pollen hydration, indicating that ADF3 acts downstream of RALF33-FER signaling. Proteomics analysis revealed that FER phosphorylates ADF3, and RALF33 treatment triggers this phosphorylation in vivo, providing a mechanistic link between RALF33–FER signaling and ADF3-mediated actin remodeling.

To investigate the effect of ADF3's phosphorylation, He et al. generated 2 ADF3 variants in the *adf3* background: one that cannot be phosphorylated and one that mimics phosphorylated ADF3. Unphosphorylated ADF3 had normal actin disassembly and faster pollen hydration, whereas the phosphorylated ADF3 had reduced actin disassembly and slower hydration, indicating that FER-mediated phosphorylation inhibits ADF3's actin-depolymerizing activity.

Finally, as PCP-Bγ facilitates pollen hydration by unlocking the RALF33–FER mediated gate (Liu et al. 2021), the authors treated papillae with PCP-Bγ and observed actin remodeling. PCP-Bγ induced earlier actin accumulation at pollen contact sites in wild-type papillae, resulting in faster pollen hydration, whereas in *adf3* mutants, actin accumulation was delayed and pollen hydration was only marginally faster, indicating that PCP-Bγ acts genetically upstream of ADF3.

Taken together, this work uncovers new components of the stigmatic gate controlling pollen hydration. Prior to pollination, FER phosphorylates ADF3 in response to stigma-derived RALF33 peptides, inhibiting ADF3's actin-depolymerizing activity to maintain a stabilized actin network in stigmatic papillae (Fig. 1b). Upon pollination, pollen-derived PCP-Bγ peptides likely relieve this restraint through FER, activating ADF3 and inducing actin remodeling and accumulation at the pollen contact site, thereby promoting pollen hydration (Fig. 1b). FER-mediated signaling modulates actin organization in stigmatic papillae through ADF3, determining whether the stigmatic gate remains closed or open to pollen hydration. By imposing a controlled checkpoint in actin remodeling, FER-mediated signaling creates a temporal window during which stigmatic papillae can evaluate pollen identity, ensuring that hydration occurs only for compatible pollen.

Despite these advances, key questions remain. How does actin remodeling in papillae promote pollen hydration: through vesicle trafficking, alterations in vacuolar structures, or other mechanisms? How does PCP-Bγ activate ADF3 in papillae, and which kinases control its depolymerization activity? Does FER regulate ADF3 solely via direct phosphorylation or also indirectly through Rho-like GTPases? What drives the directional focusing of actin at the pollen contact site, and does mechanical pressure from pollen contribute, with FER acting as a potential mechanosensor? It also remains unclear how actin dynamics are coordinated with vesicle secretion, aquaporin activation, ROS regulation, and water release, and how these multiple signaling pathways are integrated to ensure the precise timing, specificity, and directional of water delivery to pollen. Addressing these questions will be essential to understand how pollen- and stigma-derived signals coordinate actin remodeling and pollen hydration.

## Recent research articles in *Plant Physiology*:

- Lee et al. (2024) showed that Arabidopsis leucine-rich repeat malectin (LRR-MAL) receptor kinases in the stigma promote intraspecific pollen tube growth and block interspecific pollen (https://doi.org/10.1093/plphys/kiae038).
- Zhang et al. (2024) developed nontransformation methods in *Brassica rapa* to manipulate signaling pathways and genes to study pollen–stigma interactions and self-incompatibility (https://doi.org/10.1093/plphys/kiae445).

## Data Availability

No new data were generated or analyzed in support of this research.

## References

[kiag137-B1] Chapman LA, Goring DR. 2010. Pollen–pistil interactions regulating successful fertilization in the Brassicaceae. J Exp Bot. 61:1987–1999. 10.1093/jxb/erq021. 20181663

[kiag137-B2] Dresselhaus T, Franklin-Tong N. 2013. Male-female crosstalk during pollen germination, tube growth and guidance, and double fertilization. Mol Plant. 6:1018–1036. 10.1093/mp/sst061. 23571489

[kiag137-B3] He YJ et al 2025. Multiple gatekeeping steps in pollination lock species specificity. J Exp Bot. 76:1510–1523. 10.1093/jxb/erae488. 39673238

[kiag137-B4] He YJ et al 2026. Decoding pollen hydration: the role of FERONIA-mediated signaling in stigmatic actin cytoskeleton dynamics. Plant Phys:kiag117. 10.1093/plphys/kiag117. 41771674

[kiag137-B5] Huang J et al 2023. Stigma receptors control intraspecies and interspecies barriers in Brassicaceae. Nature. 614:303–308. 10.1038/s41586-022-05640-x. 36697825 PMC9908550

[kiag137-B6] Iwano M et al 2007. Actin dynamics in Papilla cells of Brassica rapa during self- and cross-pollination. Plant Physiol. 144:72–81. 10.1104/pp.106.095273. 17337527 PMC1913780

[kiag137-B7] Lee HK, Canales Sanchez LE, Bordeleau SJ, Goring DR. 2024. Arabidopsis leucine-rich repeat malectin receptor-like kinases regulate pollen-stigma interactions. Plant Physiol. 195:343–355. 10.1093/plphys/kiae038. 38270530

[kiag137-B8] Liu C et al 2021. Pollen PCP-B peptides unlock a stigma peptide–receptor kinase gating mechanism for pollination. Science. 372:171–175. 10.1126/science.abc6107. 33833120

[kiag137-B9] Lu YJ et al 2020. Arabidopsis calcium-dependent protein kinase 3 regulates actin cytoskeleton organization and immunity. Nat Commun. 11:6234. 10.1038/s41467-020-20007-4. 33277490 PMC7718926

[kiag137-B10] Rozier F et al 2020. Live-cell imaging of early events following pollen perception in self-incompatible Arabidopsis thaliana. J Exp Bot. 71:2513–2526. 10.1093/jxb/eraa008. 31943064 PMC7210763

[kiag137-B11] Wang Q et al 2023. Activation of actin-depolymerizing factor by CDPK16-mediated phosphorylation promotes actin turnover in Arabidopsis pollen tubes. PLoS Biol. 21:e3002073. 10.1371/journal.pbio.3002073. 37011088 PMC10101649

[kiag137-B12] Zhang L et al 2024. Nontransformation methods for studying signaling pathways and genes involved in Brassica rapa pollen-stigma interactions. Plant Physiol. 196:1802–1812. 10.1093/plphys/kiae445. 39213415 PMC11531837

